# Developmental Consequences of Restrictive Eating Disorders after Childhood Obesity: Two Case Reports

**DOI:** 10.51894/001c.5782

**Published:** 2017-02-02

**Authors:** Samantha Fugate Kennedy, Katherine Krive

**Affiliations:** 1 Department of Psychiatry Residency Program Michigan State University; 2 Department of Psychiatry, Assistant Program Director Michigan State University

**Keywords:** eating disorders, childhood obesity, anorexia nervosa

## Abstract

Although childhood overweight has been associated with an increased risk for later development of eating disorders, there has been little research indicating whether previously overweight children and adolescents have an increased risk for restrictive eating disorders such as anorexia nervosa or atypical anorexia nervosa. The fifth edition of the *Diagnostic and Statistical Manual of Mental Disorders* (DSM-5) defines anorexia nervosa as the restriction of energy intake relative to requirements, leading to a significantly low body weight, an intense fear of gaining weight or becoming fat, and disturbance in the way in which one’s body weight or shape is experienced. In atypical anorexia nervosa, the DSM-5 specifies that all criteria for anorexia nervosa can be met for this disorder, except that after despite significant weight loss the individual’s weight is currently within or above the normal range. In treatment of these disorders, the intense fear of weight gain may be further intensified in previously obese children because they have earlier experienced the significant consequences of obesity. The purpose of this report is to present two cases of previously obese children who later developed restrictive eating disorders. These cases demonstrate how previously obese children may be at risk for restrictive eating disorders with treatment further complicated by an intensified fear of recurrent weight gain. In these cases, treatment was complicated by an intensified fear of weight gain due to the patients’ prior obesity. The mortality rate of restrictive eating disorders may be increased in previously obese children and adolescents due to delay in diagnosis and more complicated treatment, making clinician screening imperative.

## INTRODUCTION

Childhood overweight is often associated with an increased risk for later development of eating disorders. Overweight children frequently have symptoms of excessive shape and weight concerns, binge eating and pathogenic weight loss behaviors, such as calorie restriction, excessive exercise, self-induced vomiting and misuse of laxatives or diuretics.^1,2^ Overweight children can display elevated levels of weight dissatisfaction compared to average-weight children and may utilize calorie restriction in an attempt to control their weight.^3^

Research has indicated that children and adolescents who were previously overweight are at increased risk for bulimia nervosa and binge-eating disorders.^1,2,4,5^ However, there has been little research indicating whether previously overweight children and adolescents have an increased risk for restrictive eating disorders including anorexia nervosa or atypical anorexia nervosa.^6,7^

The fifth edition of the *Diagnostic and Statistical Manual of Mental Disorders* (DSM-5) ^8^ defines anorexia nervosa as *the restriction of energy intake relative to requirements, leading to a significantly low body weight in the context of age, sex, developmental trajectory, and physical health.* Additional signs can include intense fear of gaining weight or becoming fat, persistent behaviors that interfere with weight gain, disturbances in the way in which one’s body weight or shape is experienced, undue influence of body weight or shape on self-evaluation, or lack of recognition of the seriousness of one’s current low body weight.^8^

Under the DSM-5 category of *Other Specified Feeding or Eating Disorder*, atypical anorexia nervosa is defined as meeting all the criteria for anorexia nervosa, except that despite significant weight loss, the individual’s weight is within or above the normal range.^8^ Restrictive eating disorders have been associated with higher mortality rates than bulimia nervosa and binge-eating disorder and anorexia nervosa has been shown to have the highest mortality rate of all psychiatric illnesses.^9^

The current literature regarding the treatment of restrictive eating disorders emphasizes the importance of full weight restoration through high-calorie diets.^9^ This goal may prove difficult for patients with anorexia nervosa due to their preoccupation with shape and weight and the fear of becoming obese again. This type of fear in previously obese children may also complicate treatment.

The purpose of this report is to present two cases of previously obese children who later developed restrictive eating disorders. Both patients presented to the Michigan State University Psychiatry Clinic in 2015 and were evaluated and treated by the psychiatry resident author (SFK). At the time of initial evaluation, the *short form* of the *Eating Attitudes Test* (EAT-26)^10^ was administered to both patients. The EAT-26 is a validated screening tool used to assess eating disorder risk. A score of 20 or greater on the EAT-26 indicates risk for an eating disorder.^10^

### Description of Cases

### Case A

Case A, a 15-year-old female, lived at home with both parents and an older brother. Growing up, she was described as a “big eater.” In 8^th^ grade at age 12, she was obese with weight 66.2 kg. and height 152.4 cm. Her BMI at that time was 28.5 kg/m^2^, placing her in the 96^th^ percentile for age and sex, placing her in the obese category. (For children and adolescents, BMI weight status categories are not determined as categories are in adults). The patient’s vital signs were within normal limits and she had no diagnosed medical problems. She began to slowly lose weight by cutting back her portion sizes. Toward the end of 8^th^ grade, she began to “hate” her body and developed pathogenic weight loss behaviors, including skipping lunches in order to achieve better results. This practice was soon followed by skipping breakfast as well.

With high school approaching and her concerns over her shape growing, she began purging after dinner seven nights per week. During the summer before high school, she attended a camp and was thus consistently provided higher calorie meals. In response, she began excessively exercising, once completing 1600 abdominal crunches in a single day, to counteract her “excessive” intake.

She began to explore online content regarding how to lose weight, deciding on a 500 calorie deficit as well as burning 500 calories per day through exercise. Initially, she limited herself to 1500 calories per day, but then decreased to 1000 calories per day. During a six-month period, she lost approximately 18.6 kg. and her menses ceased for three months. Due to online information, she believed she was losing weight in a healthy manner and asked to see a nutritionist who she believed would agree with her.

At that time, she was diagnosed with anorexia nervosa and was referred to the MSU Psychiatry Clinic for treatment. At the time of initial psychiatric evaluation at age 15, her weight was 45.8 kg. and height 157.5 cm. Her BMI was 17.6 kg/m^2^, placing her in the 13^th^ percentile for age and sex. In children and adolescents, significantly low weight is what is less than that minimally expected and determined by growth curves. Her EAT-26 score was 31, indicating risk for an eating disorder.

The patient’s treatment plan included weekly psychotherapy with the psychiatry resident, consisting of supportive psychotherapy and cognitive behavioral therapy (CBT), which focused on treating cognitive distortions related to her shape. Her vitals were taken weekly at these appointments, including weight which was taken with patient facing away from the scale. She saw a nutritionist once every two weeks for refeeding, which is the restoration of normal nutrition after a period of starvation. She was placed on a meal plan consisting of three meals and three daily snacks which were prepared by her parents. At time of presentation, her daily caloric intake increased gradually from her reported caloric intake to allow her body to adjust to the increased intake.

During refeeding, she experienced constipation which was successfully treated with Miralax. After four months of treatment, the patient achieved weight restoration to 54.4 kg. and height remained 157.5 cm. Her new BMI was 20.7 kg/m^2^, placing her in the 55^th^ percentile. Although the patient was able to maintain her weight, she continued to struggle with shape preoccupation and fixated on eating healthy due to her “terror” of becoming obese again. She frequently expressed concern regarding the number of calories she was required to consume. Following an additional three months of psychotherapy with an emphasis on CBT, she began to shift her value of self from shape to achievement in athletic activities and relationships with friends and family. She began to think of food as fuel for her activities, which helped to assuage her fear of becoming obese. Although her fear of becoming obese lessened, the fear remained to some extent. At time of submission, the patient continued in biweekly psychotherapy.

### Case B

Case B, a 17-year-old female, lived at home with both parents and an older sister. In 8^th^ grade at age 13, she was obese with weight 90.0 kg. and height 167.6 cm. BMI was 32.1 kg/m^2^, placing her in the 98^th^ percentile for age and sex. Her pediatrician recommended weight loss. She began to slowly lose weight by exercising and decreasing consumption of “junk” foods. Following menarche at age 15 during her sophomore year of high school, her weight loss was more difficult and she began utilizing pathogenic weight loss behaviors, including calorie counting. She began to restrict certain foods that she considered calorie-dense or foods with high fat or sugar content from her diet. This resulted in weight loss, although she became more concerned about her shape. She expressed feeling terrified of gaining the weight back and becoming obese again.

During her senior year of high school at age 17, she began to severely restrict her caloric intake. Her mother noticed her “strange” eating habits, such as eating lettuce without dressing and consuming large amounts of egg whites. She was consuming only 500 calories per day and exercising for at least 30 minutes daily. Additionally, she was using laxatives and occasionally purging to further control her weight. Due to online information she had found regarding weight loss, she felt that her eating habits were healthy.

She was referred to the MSU Psychiatry Clinic for treatment and presented with weight 58.5 kg. and height 171.4 cm. Her BMI was 19.9 kg/m^2^, placing her in the 30^th^ percentile for age and sex. The patient’s EAT-26 score was 23, indicating a clear risk for an eating disorder. Despite significant weight loss, her weight was still within normal parameters. Since all other criteria for anorexia nervosa were met, she was diagnosed with atypical anorexia nervosa.

Her treatment plan consisted of weekly psychotherapy with the psychiatry resident consisting of supportive psychotherapy and CBT. CBT focused on treating her cognitive distortions regarding food and fear of being overweight. Coping techniques were developed to decrease her behaviors such as covering mirrors to decrease excessive mirror checking. Her mother was involved by monitoring her daughter for excessive exercise. No nutritionist was involved because her weight was within the normal range and she appeared to be highly motivated to increase her caloric intake on her own.

After two months of treatment, she was able to maintain her weight and increased her daily intake to 1500 calories. This increased caloric intake was initially very difficult for her, and her fear of becoming obese intensified. Following an additional two months of CBT focused on her fear of becoming overweight, she became less preoccupied with food and was able to occasionally enjoy eating dessert foods on special occasions. The patient began to shift her value of self away from shape and instead valued her achievements in other activities such as her relationships with family members and friends. Her preoccupation with food lessened, and she was able to go out with friends. However, she continued to experience fear of becoming obese. At the time of submission, she was meeting with her psychiatrist monthly for continued psychotherapy.

## DISCUSSION

This report presents two cases in which previously obese adolescents developed restrictive eating disorders with their treatment complicated by fear of becoming obese again. Although previous research has correlated childhood obesity to later development of binge-eating disorder or bulimia nervosa, there have been few studies describing an increased risk for restrictive eating disorders.^1,4^ Overweight children are more likely to develop maladaptive body image concerns and low self-concept than average-weight peers.^9^ Additionally, overweight children are frequently teased because of their weight and may therefore be limited in typical activities of childhood, further isolating them from peers.^3^

Adolescent patients with eating disorders generally possess an intense fear of gaining weight or becoming fat, which may be further increased when a patient has suffered prior consequences of obesity. In both of these reported cases, patients experienced intense fear of becoming obese again. Since they had experienced significant consequences of obesity, their fear of weight gain appeared to be intensified. In Case A, this intensified fear was complicated treatment because it interfered with their healthy weight restoration. In both cases, fears of subsequent weight gain persisted long after the resolution of other symptoms such as restriction of energy intake and undue influence of shape on self-evaluation.

Eating disorders are typically underdiagnosed in the pediatric population, resulting in many adolescents who do not recover, or may only partially recover.^9^ Studies have demonstrated that diagnosis of eating disorders can be delayed in patients with a history of obesity.^9,11^ Clinician diagnosis may also be missed in previously obese children and adolescents because they may be normal weight even after significant weight loss.^12^ Missing or delaying the diagnosis of restrictive eating disorders may increase the potential for long-term complications in children and adolescents.

Since treatment is often more difficult in previously obese children, patients’ risk for life-threatening complications may increase even further. Eating disorders can result in a multitude of consequences for every organ system and anorexia nervosa has been shown to impose the highest mortality rate of all psychiatric illnesses.^9^ In fact, most deaths associated with anorexia nervosa are due to medical complications or suicide.^4^

As the prevalence of obesity among children and adolescents continues to increase,^13^ health professionals must remain aware that obese children and adolescents may be at increased risk for development of restrictive eating disorders following weight loss. Obese or previously obese children and adolescents presenting with weight loss, restrictive eating behaviors, frequent vomiting, excessive or compensatory exercise, or concerns about body image should be assessed for eating disorder risk.^9^ Screening tools, such as the *Children’s Eating Attitudes Test* (ChEAT-26)^14^ or the EAT-26 for adolescents,^10^ can be used to assess patients’ eating disorder risks.

## CONCLUSIONS

Although previously obese children may be at risk for development of restrictive eating disorders, there is currently little research on this potential risk. These children may be at risk for increased complications due to clinicians’ lack of suspicion resulting in delayed diagnosis. Additionally, treatment in previously obese children and adolescents may be further complicated by an intensified fear of weight gain due to the significant consequences of obesity they have previously experienced. For treatment to be effective to prevent long-term complications, early identification by clinicians is imperative.^15^

### Conflict of Interest

The authors declare no conflict of interest.
